# Local Anesthetic Lidocaine and Cancer: Insight Into Tumor Progression and Recurrence

**DOI:** 10.3389/fonc.2021.669746

**Published:** 2021-06-24

**Authors:** Caihui Zhang, Cuiyu Xie, Yao Lu

**Affiliations:** ^1^ Department of Anesthesiology, The First Affiliated Hospital of Anhui Medical University, Hefei, China; ^2^ Ambulatory Surgery Center, The First Affiliated Hospital of Anhui Medical University, Hefei, China

**Keywords:** lidocaine, cancer, metastasis, recurrence, surgery, tumor, molecular mechanisms

## Abstract

Cancer is a leading contributor to deaths worldwide. Surgery is the primary treatment for resectable cancers. Nonetheless, it also results in inflammatory response, angiogenesis, and stimulated metastasis. Local anesthetic lidocaine can directly and indirectly effect different cancers. The direct mechanisms are inhibiting proliferation and inducing apoptosis *via* regulating PI3K/AKT/mTOR and caspase-dependent Bax/Bcl2 signaling pathways or repressing cytoskeleton formation. Repression invasion, migration, and angiogenesis through influencing the activation of TNFα-dependent, Src-induced AKT/NO/ICAM and VEGF/PI3K/AKT signaling pathways. Moreover, the indirect influences are immune regulation, anti-inflammation, and postoperative pain relief. This review summarizes the latest evidence that revealed potential clinical benefits of lidocaine in cancer treatment to explore the probable molecular mechanisms and the appropriate dose.

## Introduction

Cancer remains a major cause of human death worldwide, with increasing mortality and incidence as the population ages, greatly endangering human health (1, 2). In 2017, tracheal, bronchus, lung cancer, colorectal cancer, breast cancer, and prostate cancer were the four primary reasons for cancer-related deaths in humans, which also were the main causes of cancer disability-adjusted life-years (3). Moreover, the number of deaths caused by cancer was 14 million in 2012, was estimated to reach about 9.6 million in 2018, the incidence of new cancer cases was calculated to 18.1 million in 2018 and expected to reach 24 million and 34 million by 2035 and 2050 overall global (4–6). Thus, cancer is universally considered as a great challenge and threat to global human health, with a wider social and economic burden worldwide (7). Thus, improvements in investigations and treatments are urgently needed.

Surgery is essential for global cancer therapy, especially solid organ cancers, which has a long and distinguished history. It plays a vital role in cancer prevention, diagnosis, treatment, and reconstruction (8, 9). Previous evidence indicated that more than 80% of patients with cancer may require surgery for the removal in 2015. The number of surgical procedures is expected to reach about 45 million annually worldwide by 2030 (10, 11). However, long-term cancer outcomes after surgery have not significantly improved as expectantly, conversely, tumor recurrence and metastasis may be enhanced and accelerated by surgical removal thereby causing higher mortality in comparison with the primary tumor (12, 13). Recurrence and metastatic diseases following surgical resection are reported in numerous studies, involving many molecules and elements. Demicheli and his colleagues first revealed that tumor growth may be promoted by postoperative trauma and inflammation, involving in many growth factors such as Vascular endothelial growth factor (VEGF), epidermal growth factor-like growth factors (EGFR), and endostatin (14, 15). The activation of VEGF and EGFR are crucial in postoperative wound healing *via* promoting the new angiogenesis and growth of epidermal cell, but resulting in cancer metastasis by unintentionally providing more opportunities for cancer cells to enter into vessels and enhancing their proliferation. The surgery could also open a window for the tumor cells entering into circulation known as circulating tumor cells (CTCs) by causing inflammatory response thereby finishing remote metastasis in prostate cancer (16). Tohme and colleagues demonstrated that the growth of new metastatic cancer in hepatoma was significantly enhanced for the stress after surgery *via* influencing the formation of neutrophil extracellular traps (NET) (17). The other mechanisms of tumor metastasis and recurrence after surgery are immunosuppression, gene mutations, and inflammation. These alterations in the tumor microenvironment were attributed to the harmful effects of surgery and were critical to tumor progression. Hence, the intervention of these changes may be an appropriate and significant approach to improve cancer outcomes (18, 19).

Lidocaine is commonly used in anesthesia management as one of a local anesthetics. Evidence from clinical and laboratory studies have suggested that lidocaine is beneficial to cancer patients by reducing cancer progression and recurrence and improving the survival ratio (20–22). The primary aim of this review is to document the conducive effects of using lidocaine during cancer surgery and outline the mechanisms of lidocaine inhibiting cancer invasion and metastasis.

## Lidocaine

Lidocaine is an amide local anesthetic. The analgesic efficacy of lidocaine after operation was first investigated in 1951 in intravenous administration (23). Local administration or intravenous lidocaine (IVL) leads to better airway management in the context of general anesthesia by reducing the incidence of irritating cough and sore throat (24, 25). Recently, IVL plays an increasing critical role in “day surgery” and Enhanced Recovery After Surgery programs (26, 27). Furthermore, lidocaine is one of the leading and common researched and used local anesthetics. It is routinely administered regionally for topical or surface anesthesia, injection into sub-arachnoid space and epidural space for blocking the local motor and sensory nerves (28, 29). All of these are the reasons why we choose to investigate lidocaine, and our main purpose is to review the beneficial effects of lidocaine on cancer.

## Lidocaine and Cancer

**Figure 1:**
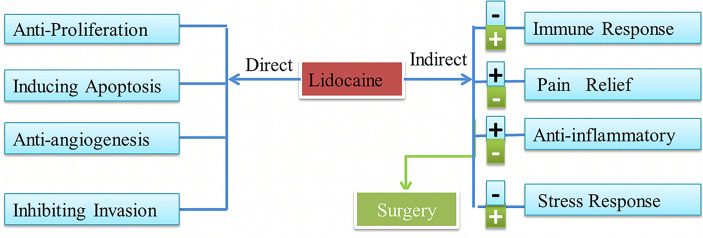
Lidocaine has direct and indirect effects on cancer and the influences of the surgery. Lidocaine also can inhibit the negative attack of surgery.

Thus far, several retrospective studies have found that lidocaine has indirect effects and direct effects on tumor progression. These effects are shown in **Figure 1**.

### Indirect Effect

Lidocaine can influence the tumor microenvironment by regulating the immune and inflammatory response, alleviating the pain of surgery, and modulating the response of the neuroendocrine system. The *in vitro* study of Ramirez and colleagues showed that the function of natural killer (NK) cells can be enhanced by clinical concentrations of lidocaine *via* regulating the release of lytic granules. NK cells are one of the crucial elements of the anti-tumor immune response (30). Furthermore, in different phases of tumor development, immune and inflammatory responses play pivotal roles, including initiation, progression, malignant transformation, invasion, and metastasis (31). More importantly, Piegeler and colleagues demonstrated that lidocaine blocked metalloproteinase-9 (MMP-9) release by suppressing Src-dependent inflammatory signaling pathway at concentrations of 10 µM *in vitro* (32, 33). A review summarized that the inflammatory response in tumorigenesis included three parts (34):

i. Immune cells: (e.g. NK cells and neutrophils).
ii. Inflammatory entities: [e.g. cytokines, growth factors, and interleukin-6 (IL-6)].
iii. Inflammatory tumor microenvironment (e.g. fibroblasts, myeloid cells, and endothelium of new blood vessels).

Altogether, if lidocaine does have antitumor effects, its anti-inflammatory properties may have a greater effect on this process *via* influencing inflammatory cells, entities, and microenvironment. Although the underlying mechanisms require further investigation, previous studies have shown that these inflammation-driven changes and immune responses resemble alterations after the surgery of tumor removal that significantly contribute to tumor growth and progression (34, 35), which are suppressed by different concentrations of lidocaine. Moreover, lidocaine can also relieve pain, reduce surgical strikes, and alleviate the stress response in different cancers.

### Direct Effect

**Figure 2:**
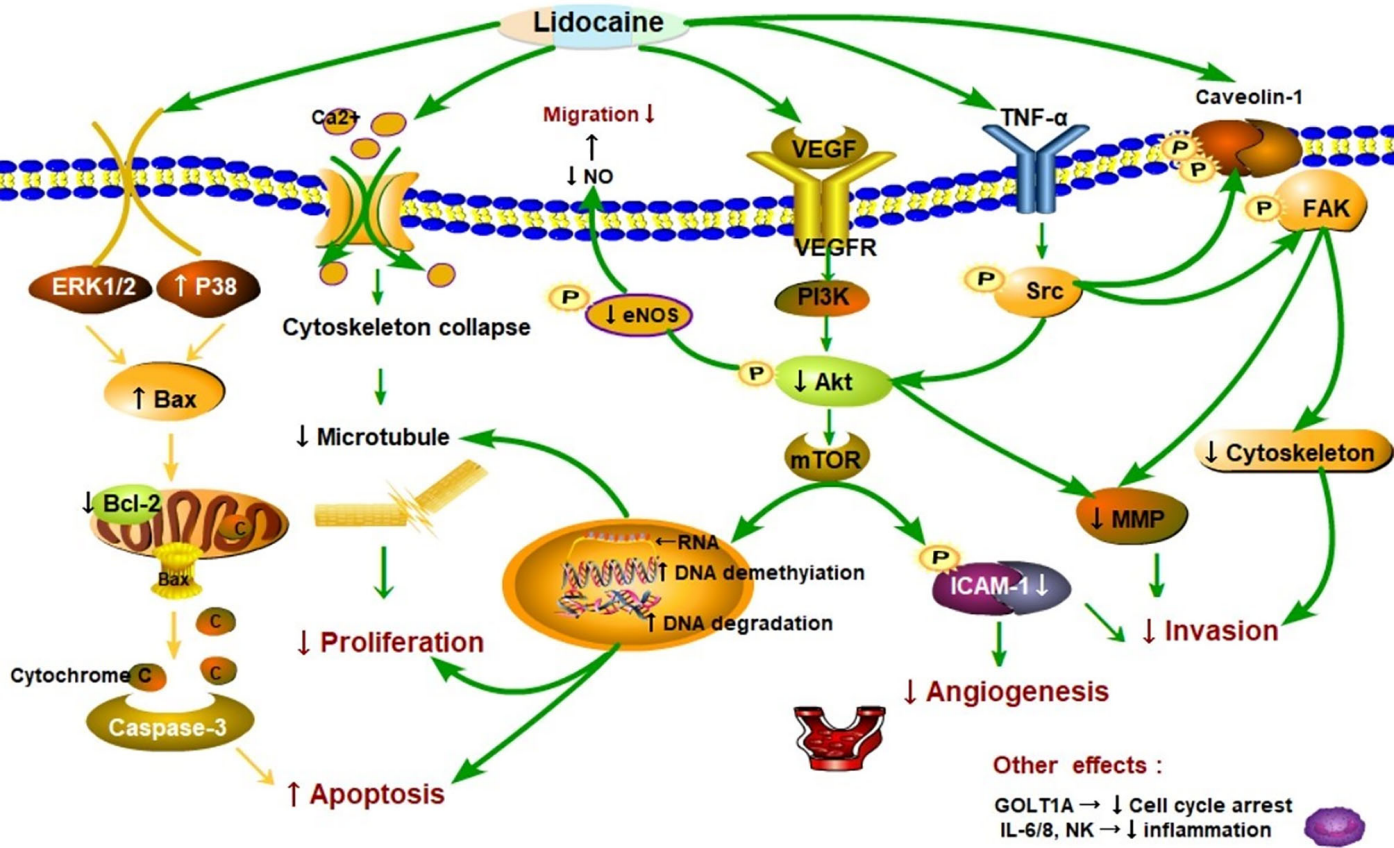
Schematic representation of the proposed mechanisms that lidocaine suppresses proliferation, migration, and induces apoptosis in cancer progression. As shown in the picture, lidocaine blocks tumor necrosis factor α (TNF-α)–dependent activity of Src tyrosine protein kinase (Src) *via* repressing function of TNF receptor 1 (TNF-R1), thereby preventing Akt kinase (Akt) and focal adhesion kinase (FAK) from activating caveolin-1 from phosphorylating (33). Moreover, lidocaine inhibited the Src-dependent intercellular adhesion molecule-1 (ICAM-1) phosphorylation to block the cancer cells adhesion and invasion (38). These signaling subways play a critical role in cancer metastasis. Cancer cells can migrate to remote sites *via* breaking up the cytoskeletal structure [e.g. microtubules] and releasing of matrix-metalloproteinases (MMP) (39). Lidocaine impairs cancer cells proliferation and cytoskeletal reorganization by acting on DNA methylation and repressing the (vascular endothelial growth factor) VEGF/AKT/mTOR signaling pathways (40–42). Endothelial nitric oxide synthase (eNOS) and nitric oxide (NO) generation was also inhibited by lidocaine thereby reducing vascular dilatation and directly decreasing the cells migration (38). Lidocaine depressed tumor angiogenesis *via* suppressing the VEGF/AKT/mTOR/ICAM-1 signaling pathway (43, 44). Consequently, AKT related signaling pathways were essential in cancer metastasis. Additionally, lidocaine may induce apoptosis by promoting caspase-3 production by up-regulating the Bax and decreasing of Bcl-2 associating with the signaling pathways of ERK1/2 and p38 (22). In particular, lidocaine may aggravate the apoptosis through directly inhibited PI3K/AKT/mTOR or influenced AKT/Bcl2/Bax signaling pathway (42, 45, 46). The other effects are related to molecules such as natural killer cells (NK) (22).

Lidocaine suppresses tumor cell proliferation by acting negatively impacts on cancer cell signaling and modification of genes. A number of retrospective studies found that lidocaine inhibits the process of proliferation, the ability of invasion, migration, and induce apoptosis in several cancers (22, 36, 37). A schematic diagram of the possible mechanisms is shown in **Figure 2**. The study of Sun showed that the proliferation of lung cancer cells could be inhibited *via* regulation of miR-539/EGFR axis and decrease the activation of ERK and PI3K/AKT pathways (41). Moreover, lidocaine may induce apoptosis by promoting caspase-3 production *via* up-regulating the Bax and decreasing Bcl-2 associated with the signaling pathways of ERK1/2 and p38 (22). Lidocaine blocks tumor necrosis factor α (TNF-α)–dependent activity of tyrosine protein kinase (Src) *via* repressing function of TNF receptor 1 (TNF-R1), thereby preventing Akt and focal adhesion kinase (FAK) from activating, caveolin-1 from phosphorylating (33).

These signaling pathways play a critical role in cancer metastasis (**Figure 2**). Additionally, cancer cells can migrate to remote sites *via* breaking up the cytoskeletal structure [e.g. microtubules] and decreasing release of matrix-metalloproteinases (MMP) (39). The other effects are related to molecules such as vascular endothelial growth factor (VEGF), interleukin 6, 8 (IL6, IL8), and Golgi transport 1A (GOLT1A). The proliferation of lung cancer cells can be repressed by lidocaine *via* decreasing GOLT1A generation (37). In another study, Lirk and his colleagues examined that the methylation of cancer cell deoxyribonucleic acid (DNA) was inhibited by lidocaine (40). This alteration results in the re-expression of the previously hypermethylated silenced genes (e.g. tumor suppressor genes) and repression of cancer formation. Piegeler and colleagues reported that lidocaine reduced tumor cell ability of invasion by repressing MMP-9 formation and release (33). The extracellular matrix and the basal lamina can be degrade and broken up by MMP-9, thereby the cancer cells will finish invasion and remote metastasis (47, 48). In a word, lidocaine inhibits the process of proliferation, suppresses the capabilities of invasion and migration, and induces apoptosis in several cancers.

## Mechanisms of Cancer Metastasis

The metastatic spread of cancer cells to distant anatomical locations is a more common cause of cancer-related death compared with primary tumor in malignant tumor (49). In addition, invasion and metastasis are the marked characteristics of a malignant tumor and also the major reasons for poor prognosis in clinical therapy (12). The three primary pathways for tumor metastasis are hematogenous, lymphatic, and implant metastasis. The potential mechanisms and relative components of tumor metastasis and recurrence are the following (50–52):

(e.g., hematogenous metastasis)

i. The adhesion between cancer cells is weakened (cell adhesion molecules, integrin family, selectin family).
ii. The cancer cells attach to the basement membrane (integrin families, β1, β2).
iii. Basement membrane degradation (fibrinolytic enzyme activators, cathepsin D, MMP, AKT).
iv. Cancer cells intravasate into blood vessels (autocrine migration factor, FAK, growth factor).

The process of cancer invasion and metastasis includes a series of steps, for example, tumor growth *in situ*, invasion of surrounding tissues, contact with small-vessel walls, tumor cells entering blood circulation, and growing in distant organs, in which the tumor cells entering into blood circulation and extracellular matrix (ECM) degradation are of great clinical significance (53, 54). Currently, numerous studies have revealed that Akt-dependent signaling plays a crucial role in tumor invasion and functions as a potent pro-metastatic mechanism. The study of Tian and colleagues showed that AKT-induced lncRNA VAL promotes EMT-independent metastasis *via* reducing Trim16-dependent Vimentin degradation (55). Moreover, PI3K/Akt pathway was demonstrated as a therapeutic target in breast cancer associated with tumor suppressor miRNA-204-5p (56). AKT excessive activation participates in many signaling pathways *via* regulation the expression oncogenes and tumor suppressors, such as EGFR, Ras, PI3K, BRAF, AKT itself, and natural AKT inhibitor PTEN, and is of great importance during tumor metastases and progression (57, 58). As for ECM degradation, lysosomes have been considered a pivotal element in tumor invasion and metastasis as well. It could facilitate tumor cell migration and invasion *via* secreting acid hydrolase thereby increasing matrix remodeling (59). Lysosomes also promote cell adhesion and influence integrin secretion through attaching to ECM and regulating the dynamics of focal adhesions, activating AKT inducing cancer and metastasis (60–62). Taken together, the process is tanglesome, but has great therapeutic potential, such repression by the lidocaine, intervention by the stress of surgery.

## Lidocaine and Surgery

Surgery is the foremost treatment strategy for the majority of patients with solid tumors. Also, it is a severe attack on human, resulting in immunosuppression, angiogenesis, inflammatory response, and stimulating pain. Moreover, the more significant of the surgery the greater the surgical stress response. It is considered that surgical stress contributes to cell mediated immune system (CMI) suppression and promotes tumor progression (63). Radical treatment of cancers may inadvertently provide malignant cancer cells with chances to break down the host barriers and to form remote metastatic tumors that may denote poor prognosis (64). Surgery modulates and induces tumor metastasis, but lidocaine can inhibit activity and chemotaxis of leukocyte to the sites of surgical incision both in animal and clinical studies, which is also associated with damping of the surgery-induced generation of inflammatory cytokines (34, 65, 66). Therefore, the perioperative use of lidocaine may improve the outcomes of cancer patients by decreasing surgical strike as showed in **Figure 1**. At the same time, studies have shown that lidocaine can inhibit inflammation, analgesia, and angiogenesis, which can be associated with indirect effects on cancer and the negative changes after cancer surgery.

## Lidocaine Indirect Influence

### Repression of Immune and Inflammatory Response

Numerous researches have demonstrated the capabilities of lidocaine that could repress the inflammatory response by obstructing the secretion of inflammatory mediator and down-regulating the activation of immune cells, such as macrophages, NK cells, and neutrophils (67, 68). Studies that may predict the anti-inflammatory effects of lidocaine are shown in **Table 1**. They can be divided into laboratory studies and clinical studies.

**Table 1:** The indirect effects of lidocaine repress the immune and inflammatory response.

Study	Year	Materials	Studied concentrations	Mechanisms	Results
Piegeler and colleagues (69)	2012	Cancer cells	(1 nM–100 muM)	Src, TNF-α	Lidocaine combined with TNF-α significantly decreased inflammatory Src-activation and ICAM-1 phosphorylation may provide beneficial antimetastatic effects.
Piegeler and colleagues (33)	2015	Cancer cells	(1–100 µM)	TNF-α, Akt, FAK, MMP	Lidocaine blocked tumor cells invasion and MMP-9 and FAK secretion by attenuating Src-dependent inflammatory signaling pathways.
Chiu and colleagues (70)	2016	Sprague–Dawley rats	(0.8 or 4 mg/kg, i.p.)	IL-1, 6 and TNF-α	Lidocaine pretreatment decreased the release of IL-1β, IL6, and TNF-α to repress the inflammatory response.
Lin and Colleagues (71)	2020	C57BL/6 Mice	(1 to 10 μM)	HIF1-α, and IL-6 TNF-α	Lidocaine reduces the release of TNF-α and IL-6 and inhibits the HIF-1α induced inflammatory cascades.
Galos and colleagues (72)	2020	Women (n = 120)	(1, 1.5, 2 mg kg1)	NETosis and MMP-3	I.V. perioperative lidocaine might reduce postoperative recurrence related to decreasing the expression of NETosis and MMP3.

TNF-α, tumor necrosis factor α; ICAM-1, intercellular adhesion molecule-1; FAK, focal adhesion kinase; MMP, matrix-metalloproteinases; HIF-1α, Hypoxia Inducible Factor-1a; IL, interleukin 6; Src, Src tyrosine protein kinase.

#### Laboratory Studies

Lidocaine inhibits immune cell activation and adhesion in both *in vitro* and *in vivo* models to the site of injury. The invasion ability of cancer cells could be intercepted by lidocaine, which was related to the decrease of TNF-α. Piegeler and his colleagues showed that clinically relevant concentrations of lidocaine significantly restricted the TNFα-dependent inflammatory response by reducing activating of Src and phosphorylation of MAPK in lung cancer cells (69). Moreover, lidocaine decreased tumor cells metastasis and MMP-9 formation and release by down-regulating Src-induced immune and inflammatory signaling pathway (33). Ramirez and his colleagues showed that NK cells activation *in vitro* was promoted by lidocaine at clinical concentration in changing the secretions of lytic granules (30). Although investigated completely in laboratory, these research results state meaningful insights into the mechanism associated with immune and inflammatory response by which local anesthetics, such as lidocaine, might diminish metastasis.

A study using an animal model also showed that lidocaine treatment preoperative could reduce microglias initiation and genes modification of pro-inflammatory factors, and lidocaine systemic administration decreased the dead and dying neurons in the hippocampus area (70). Another study indicated that inflammatory cytokines secretions were inhibited by lidocaine in a dose-dependent manner, thereby providing protection of anti-inflammatory for mice (71). In a murine model, Johnson and colleagues (73) found that compared with general anesthesia alone, the combination of lidocaine and sevoflurane anesthesia could effectively reduce postoperative metastasis of lung cancer by inhibiting inflammatory response, angiogenesis, and surgical stress. Hence, lidocaine, whether used alone or in combination with sevoflurane, may certainly have beneficial effects on reducing cancer metastasis. The anesthesia method of combining lidocaine local administration and sevoflurane anesthesia inhalation is also commonly used in clinical practice. Furthermore, these animal studies may provide significant insights for clinical studies. All these previous evidence suggest that lidocaine, which is capable of attenuating the activation of immune cells, e.g., microglia and the production of inflammatory factor, e.g. IL-1β, TNF-α, thereby decreasing tumor growth.

#### Clinical Studies

The study of Galos and colleagues revealed that the perioperative use of lidocaine reduced the surgically stimulated activation of NETosis and secretion of MMP3 (72). The consequence explains the underlying mechanisms that IVL might decrease the invasion and metastasis after surgery. NETosis may enhance cancer cell growth and invasion, resulting in breast cancer recurrences, poor prognosis, and thrombosis (74). In conclusion, lidocaine can depress immune and inflammatory response by influencing immune cells (e.g., NK cells and macrophages), reducing the release of inflammatory entities (e.g., CA3 and IL-6), inhibiting signaling pathways (e.g., Src-dependent inflammatory signaling), and damaging the inflammatory tumor microenvironment, thereby reducing and inhibiting cancer metastasis. As the number of relevant studies, as well as clinical studies on the underlying mechanisms, is limited, further research is needed.

### Alleviating the Pain of Surgery and Cancer

Clinical studies and a few laboratory studies have investigated the analgesic effects of lidocaine that are shown in **Table 2**. Systemic use of lidocaine has been observed to exhibit analgesic effects by promoting postoperation pain relief (84) and regulating the function of the central nervous system (77). Kawamata and colleagues showed that the incision-induced hyperalgesia in human skin can be reduced by treatment with lidocaine *via* suppressing the formation of superabundant pain inputs and the function of peripheral and central nerves (89). One of the most significant effects of lidocaine was analgesic effect during tumor removal and the management of cancer pain, including acute stimulations and dull pain, for the intervention of hyperpathia and the production of pain inputs. Recently, clinical studies have shown that the administration method of injecting lidocaine into epidural spaces or subcutaneous tissues was safe in controlling pain of cancer and could be effectively applied to clinical practice (82). Moreover, treatment with lidocaine, including local administration or intravenous injection, is beneficial and effective for dull pain (76), neuropathic pain (78), and acute pain (90). Nonetheless, the specific mechanism of perioperative use of lidocaine for intervention the generation and propagation of pain impulses remains unclear.

**Table 2:** The indirect effects of lidocaine, alleviating the pain of surgery and cancer.

Study	Year	Sample size	Studied concentrations (mg/kg)	Results
Kang and colleagues (75)	2012	48	1.5 mg/kg	Intraoperative IV low-dose lidocaine infusion enhanced pain relief after gastrectomy for stomach cancer in men
Waraya and colleagues (76)	2012	5	Not stated	Lidocaine is effective for dull pain of skin metastases of breast cancer patients.
Kern and colleagues (77)	2013	68	5% lidocaine plaster	Treatment with 5% lidocaine medicated plaster was successful in reducing the neuropathic components or trigeminal neuropathic pain of cancer patients.
Lopez, Ramirez E (78)	2013	83	Lidocaine 5% patches	Cancer patients with NP are successfully managed with topical lidocaine 5% patch, alone or in combination with other drugs.
Garzon-Rodriguez and colleagues (79)	2013	20	lidocaine 5% patches	Lidocaine 5% patches is effective in the short-term for the treatment of neuropathic cancer pain accompanied by allodynia.
Salas and Colleagues (80)	2014	200	5–8 mg/kg	Pain success is classified as a 30% decrease in the pain level between T0 and T1 (10% of patients lost to follow-up expected) by IV lidocaine.
Gibbons and colleagues (81)	2016	4	15–50 μg/kg/min	Continuous lidocaine infusions may be an effective therapy for a more diverse array of refractory cancer pain in pediatric oncology patients.
Seah and colleagues (82)	2017	20	0.67 mg/kg/h	Subcutaneous lidocaine infusions decreased in pain scores in cancer patients and had no relevant documented adverse events.
Kendall and colleagues (83)	2018	148	1.5	IV lidocaine reduced the incidence of postsurgical pain at rest at 6 months in patients undergoing breast cancer surgery.
Khan and colleagues (84)	2019	100	1.5	Perioperative lidocaine infusion may reduce persistent NP after breast cancer surgery.
Lee and colleagues (85)	2019	60	4–5	Lidocaine infusion of over 30–80 min compared with placebo for >50% reduction in cancer pain.
Omar and colleagues (86)	2019	128	1.5–2	Intravenous lidocaine can lead to reduction of opioid consumption and chronic postsurgical pain for certain types of surgery.
Hasuo and colleagues (87)	2020	25	10% lidocaine	Lidocaine ointment 10% can alleviate allodynia for 2 to 8 h after application.
Toner and colleagues (88)	2021	150	1.3–1.5	Peri-operative lidocaine infusion was safe and effective for chronic postoperative pain in breast cancer patients.

PVB, paravertebral block; NP, neuropathic pain; IV, intravenous.

Several retrospective and randomized control trials have indicated that the intravenous or local use of lidocaine can lead to opioid saving and chronic postsurgical pain reduction for certain types of surgery, a reduced incidence of pain, and enhanced analgesic effects in cancer patients (83, 84). An *in vitro* study (90) demonstrated that the activities of voltage-gated sodium channels, calcium, potassium channels, and N-methyl-D-aspartic acid receptors may be inhibited by lidocaine at a low concentration, and be blocked by high concentration lidocaine. Therefore, lidocaine at different concentrations can affect transmission of pain-related electrical signals by inhibiting the activation of the relative ion channels and the function of their receptors.

Pain is common in cancer patients and considerably impairs their quality of life (91). About 30–90% of cancer patients suffer from pain, including neuropathic pain (92). Lidocaine is routinely administered regionally for topical or surface anesthesia, injection into sub-arachnoid space and epidural space to block the local motor and sensory nerves. The two ways were also applied to clinical treatment for intention of pain relief (77, 93). The intra-arterial administration of lidocaine is safely and effectively used for prevention and reduction of pain in perioperative period and results from transarterial chemoembolization of hepatocellular carcinoma (94–96). Khan and colleagues (84) showed that lidocaine persistently reduced the generation and enlargement of NP (43.1 *vs* 63.3%; relative risk = .68; 95% confidence interval = .47–1.0) in patients with breast cancer needing surgical treatment.

Overall, lidocaine exhibits analgesic, anti-inflammatory, and anti-hyperalgesic properties in cancer pain management. The pain-relieving and anti-inflammatory properties of lidocaine have been proven. Several studies also have shown that lidocaine could attenuate the inflammatory response for postoperative pain of surgery and long time NP of cancer, decrease the risk of cancer metastasis, and modulate the neuroendocrine stress response. All of these are indirect effects of lidocaine on cancers.

## Direct Influence of Lidocaine

### Suppressing Cancer Cell Proliferation and Inducing Apoptosis

#### Laboratory Studies *In Vitro*

**Table 3** shows that lidocaine suppresses tumor cell proliferation though the effects on cell signaling and influences the cell cycle and the demethylation of DNA or other genes. Chang and colleagues indicated that the cell viability and colony formation in thyroid cancer cells were suppressed by lidocaine (98). Moreover, lidocaine induced tumor cell apoptosis *via* dose-dependent manners by destroying potential of mitochondrial membrane and decreasing the release of cytochrome c. This effect is closely associated with cell signaling of p38 mitogen-activated protein kinase (MAPK) and extracellular signal-regulated kinase 1/2 (ERK1/2), along with the production of caspase-3 and -7, and reaction of a higher ratio of Bax/Bcl-2. The signal pathway is shown in **Figure 2**. Ye and colleagues (112) also showed that the activation of Bcl-2 was down-regulated and the level of Bax was increased by lidocaine treatment, so Bax/Bcl-2 may be a potential mechanism of apoptosis. In addition, lidocaine suppressed the growth of HepG2 cells, interrupted cell proliferation cycle, resulted in programmed cell death with the increase of Bax protein, and promoted caspase-3 and an accompanying reduction with Bcl-2 protein in dose-dependent and time-dependent manners (22). In particular, lidocaine may aggravate the apoptosis through directly inhibited PI3K/AKT/mTOR or indirectly influenced the AKT/Bcl2/Bax signaling pathway (45, 46). By affecting these signaling pathways, lidocaine can affect the growth, metabolism, and cytoskeleton formation of cancer cells (39) thereby repressing cells’ ability of proliferation and resulting in caspase-mediated cell death.

**Table 3:** The direct effects of lidocaine, suppressing the tumor cell proliferation, invasion and inducing apoptosis.

Study *in vitro*	Year		Materials	Studied concentrations	Mechanism	Results
Lirk and colleagues (36)	2012		Breast cancer cell lines	1, 0.01, 0.01 mM	DNA	Lidocaine time- and dose-dependently demethylates DNA of breast cancer cells.
Lucchinetti and colleagues (97)	2012		Mesenchymal stem cells	10, 100, 500 µM	Lysosome	Lidocaine reduced MSC proliferation at 100 muM may be related to cell cycle delay or the G0/1-S phase transition arrest.
Chang and colleagues (98)	2014		Thyroid cancer cells	0, 2, 4, 8, 10, 12, 14, 16 mM	MAPK/ERK, caspase 3, Bax/Bcl-2	Lidocaine reduced cancer cells viability and colony formation, induced apoptosis and necrosis in high concentrations.
Li, K. and colleagues (99)	2014		Human breast cancer lines	0.01, 0.1, 1 mM	RARbeta2, RASSF1A	Treatment with lidocaine induced cancer cells apoptosis *via* down-regulation of the expression RARbeta2 or RASSF1A.
Jiang and Colleagues (100)	2016		Human breast, prostatic Cancer cells	0, 10, and 100 µM, 1, 2, 5, and 10 mM	TRPV6	Lidocaine inhibits the invasion and migration of TRPV6-expressing cancer cells by TRPV6 down-regulation
Zhang and colleagues (37)	2017		Human lung adenocarcinoma cells	0, 0.5, 2, 8 mmol/L	GOLT1A	Lidocaine inhibits the proliferation of lung cancer cells *via* repressing the GOLT1A expression.
Xing and Colleagues (22)	2017		HepG2 cells	0.1, 0.5, 1, 2, 5, 10 mM	Caspase-3, Bcl-2, Bax, ERK1/2, P38	Lidocaine inhibited the growth of HepG2 cells in a dose- and time-dependent manner by increasing Bax protein and activating caspase-3 and decreasing Bcl-2 protein *via* the ERK 1/2 and p38 pathways.
Jurj and colleagues (101)	2017		Human hepatocarcinoma cells.	0.5, 0.75, 1, 1.5, 1.75, 2, 2.5, 3 µM	P53	Lidocaine had antiproliferative effects on human hepatocarcinoma cells, possibly by modifying the P53 expression level.
Yang and colleagues (102)	2018		Human bladder cancer cells	1.25, 2.5, or 5 mg/ml	Not stated	Lidocaine (1.25 to 5 mg/ml) repressed the proliferation of cancer cells and enhanced the actions of antiproliferative agents
Qu and colleagues (103)	2018		Colorectal cancer cells	500 muM,1,000 muM	MiR-520a-3p EGFR	Lidocaine 500 and 1,000 muM over 24 h inhibited proliferation and induced apoptosis of CRC *via* targeting EGFR
Yang and colleagues (104)	2018		Gastric cancer cells	10, 100, and 1 mM	ERK1/2	Lidocaine at (10 muM) inhibited the proliferation of cancer cells by repressing p-ERK1/2.
Chamaraux-Tran and colleagues (105)	2018		Human breast cancer cells	0.1, 0.5, 1, 5 and 10 mM	Not stated	Lidocaine reduced the viability and migration of cancer cells.
D’Agostino and colleagues (39)	2018		Human breast cancer cells	10, 100 mM	CXCR4, CXCL12	Lidocaine inhibited cancer progression and metastasis *via* blocking the signaling of chemokine CXCL12 and the activation of its receptor CXCR4.
Tat and colleagues (106)	2019		Colon cancer cell	2–4 microM	Caspase-8, HSP-27/60, IGF-II	Lidocaine repressed significantly cancer cells proliferation *via* activation of apoptosis protein pathways.
Zhu and colleagues (107)	2019		Cervical cancer cells	50, 100, 500, 1,000 µM	lncRNA-MEG3, miR-421, BTG1	Lidocaine suppressed proliferation and induced cell apoptosis of cervical cancer cells by modulating the genes expression of lncRNA-MEG3/BTG1.
Sun and colleagues (41)	2019		Lung cancer cells	8 mM	ERK, PI3K/AKT pathways	Lidocaine decreased the viability, migration, and invasion and induced apoptosis of cancer cells by increasing the expression of miR-539 and regulating the activation of ERK and PI3K/AKT pathways.
Siekmann and colleagues (108)	2019		Colon cancer cells SW480, SW620	5–1,000 µM	MMP-9	Cell proliferation was significantly reduced by 1,000 microM lidocaine.
Freeman and colleagues (109)	2019		BALB/c mice(n = 72)	1.5–2.0 mg.kg−1	Not stated	In a murine model of breast cancer surgery, lidocaine decreased pulmonary remote metastasis.
Wall and colleagues (110)	2019		BALB/c mice (n = 95)	1.5, 2.0, 5.0 mg.kg−1	MMP-2/9, Src	Lidocaine reduced lung metastasis *via* effecting on release of MMP-2 and MMP-9 may through the Src pathways.
Johnson and colleagues (73)		2018	BALB/c mice(n = 88)	1.5, 2.0 mg.kg−1	Not stated	Lidocaine reduced lung metastatic colony count, may *via* anti-inflammatory and anti-angiogenic effects.
Freeman and colleagues (111)		2018	BALB/c mice (n = 45)	1.5, 2.0 mg.kg−1	Not stated	The combination with lidocaine and cisplatin significantly decreased metastatic lung colony count compared to control and cisplatin alone.
Yang and colleagues (104)		2018	BALB/c mice (n = 40)	1.5, 2.5, 5 mg.ml−1	Not stated	The combination of 0.66 mg/ml mitomycin C and 5 mg/ml lidocaine prolonged tumor-bearing mice survival and reduced bladder wet weight (p < 0.05).
Chamaraux-Tran and colleagues (105)		2018	SCID mice(n = 20)	8.0 mg.kg−1	Not stated	Intraperitoneal lidocaine with a clinical effected dose improved survival of mice with MDA-MB-231 peritoneal carcinomatosis.
Xing and colleagues (22)		2017	BALB/c mice(n = 32)	30 mg.kg−1, twice a week	Not stated	Lidocaine induces caspase-dependent apoptosis and suppresses tumor growth, and enhances the cytotoxicity of cisplatin, thereby inhibiting HepG2 tumor growth.

HSP, heat-shock proteins; IGF, insulin growth factor; lncRNA-MEG3, Long non-coding RNA maternally expressed gene 3 BTG1, anti-proliferation factor 1; ERK, extracellular signal-related kinases; EGFR, epidermal growth factor receptor; PI3K, phosphoinositide 3-kinase; AKT, serine/threonine protein kinase; CRC, colorectal cancer cells; TBEC, tumor breast epithelial cells; NBEC, normal breast epithelial cells; LAD, human lung adenocarcinoma; GOLT1A, Golgi transport 1A; TRPV, transient receptor potential cation channel subfamily V member 6; MAPK, mitogen-activated protein kinase; MSC, Mesenchymal stem cells; HepG2, Hepatocellular Carcinoma Cells; SCID,severe combined immunodeficiency.

With regard to the changes of genes, Lirk and colleagues demonstrated that clinically relevant concentrations of lidocaine may demethylate the DNA of breast cancer cell lines *in vitro* (36). Lidocaine represses the activation of ERK and PI3K/AKT pathways by elevating the expression of miR-539 (41). It also strengthens the cytotoxicity of cisplatin by increasing the expression of RARbeta2 and RASSF1A demethylation *in vitro* study (99). Moreover, the expression of tumor suppressor gene IncRNA-MEG3 and oncogenes miR-421 were intervened by lidocaine thereby inhibiting cervical cancer cell proliferation and induces cell apoptosis (107). In malignancy, decreased methylation generally contributes to the up-regulation of tumor suppressor genes, inhibition cancer development. Therefore, these modifications caused by lidocaine mean reduction in methylation that may increase the re-expression of tumor suppressor genes and restrain cancer progression.

#### Laboratory Studies *In Vivo*

The increase of tumor in size and weight was inhibited by lidocaine *in vivo* by repressing cancer cells proliferation, sensitizing the cytotoxic chemotherapy drugs, and inducing programmed cell death (22). Moreover, previous study demonstrated that injecting clinical concentration of lidocaine into intraperitoneal is probably able to improve the prognosis of mice with breast cancer models, resulting in the increase of survival number (105). In animal research of cancer surgery, the remote metastasis such as lung of breast cancer could be reduced by lidocaine, which may be related to the reduction of MMP-2 and MMP-9 (109, 110). MMP-9 is a critical molecule in cancer development. Recently, a study also found that the combination administration of lidocaine and cisplatin can markedly induce caspase-mediated cell death of MCF-7 cells compared with the use of cisplatin alone.

Finally, lidocaine inhibits tumor cell proliferation by affecting the cancer cell signaling and influences the cell cycle and demethylation of DNA or other genes *in vivo*. Moreover, it enhances the cytotoxicity of drugs, such as cisplatin, and decreases metastasis by reducing MMP-2 and MMP-9 activation *in vivo*; moreover, relevant discoveries and research in clinical practice are still needed.

### Inhibiting the Process of Invasion

The process of invasion is extremely complicated, which consists of a series of steps, including basement membrane degradation and invasion of surrounding tissues, contacting with small-vessel walls, and entering of tumor cells into the circulatory system. One of the most studied is basement membrane degradation. Piegeler and colleagues (33) showed that lidocaine dramatically inhibited the TNFα-dependent signaling pathway by avoiding activating/phosphorylation of Akt, FAK, and caveolin-1 (Cav1), thus attenuating MMP release and invasion in NCI-H838 cells. FAK also plays a crucial role in the process of cancer cells remote movement, involving in orienting appropriate sites, assembling/reorganizing of actin cytoskeletal, and eventually migrating of cancer cells. Cav1 is a fundamental element of construction of caveolae, which is related to adhesion and invasion of cancer cells (33, 113, 114).

The malignant cells may have analogous characteristics of secreting MMPs to neutrophils, the invasion and migration of cancer cells are enhanced and basal lamina as well as the extracellular matrix are disintegrated by MMPs, which provides tumor cells with an opportunity entering into circulatory system and finish the distant metastasis (115). Wall and colleagues (110) also demonstrated that lidocaine reduced MMP-9 and MMP-2 activities *via* an inhibitory effect on the Src pathway in an *in vivo* model. Furthermore, the study of Zhang and colleagues (116) reported that lidocaine inhibited the invasion and migration of cancer cells *via* down-regulating the AKT/mTOR and β-catenin pathway. The AKT/mTOR pathway was demonstrated closely associated with the activation of lysosome, the release of hydrolase, the degradation of the ECM (59, 117).

Overall, with the use of lidocaine, inhibiting the signaling pathways is perhaps an effective way of repressing the invasion of malignant cells. Furthermore, these findings can provide significant insights for further clinical studies by which lidocaine might decrease invasion and metastasis.

### Depressing Tumor Angiogenesis

Angiogenesis plays a vital role in tumorigenesis and metastasis, which provides sufficient nutrient substances as well as oxygen for cancer cells, efficiently drains the metabolic waste, supplies more opportunities for cancer cell attaining remote migration (118). Moreover, the level of tumor vascularization is closely connected to the development of hematogenous metastasis as well as tumor grade (119). Previous studies have demonstrated that the VEGF and its receptor were significantly critical in angiogenesis through the AKT/PI3K signaling pathways to up-regulation of ICAM-1 eventually (120). The up-regulation ICAM-1 indicates more cancer cells and endothelial cells adhesion and migration associated with activation of VEGF and AKT/PI3K signaling pathways (121). The tumor growth and progression require angiogenesis, which inhibitions are a crucial therapeutic strategy for cancer patients. Hence, Lan and colleagues (122) (123) reported that the expression of endothelial ICAM-1 was reduced by lidocaine treatment, especially when concentrations higher than on clinically effective blood concentrations. Furthermore, the activation of VEGF-A and phosphorylation VEGFR-2 were suppressed by lidocaine, thereby decreasing the number and degree of angiogenesis on a clinically relevant concentrations without causing cell death (124). Lidocaine also inhibits endothelial cell capillary network for construction and VEGF, decreases endothelial cells to migration and propagation by interfering in the preliminary period of the formation of new blood vessels *in vitro* (43). Additionally, lidocaine has been reported to significantly suppress the activation of ERK and PI3K/AKT pathways (41), which are essential in VEGF secretion, eNOS phosphorylation, vasorelaxation, and angiogenesis (125). The angiogenic ability of vascular endothelial cells could be pathologically enhanced by VEGF. Moreover, VEGF as well as its receptor (VEGFR) are of great significance in anti-angiogenesis treatment (126). These findings could provide more evidence on the ability of lidocaine to inhibit cancer metastasis *via* repressing angiogenesis. Moreover, the underlying mechanism is partly associated with down-regulation of PI3K/AKT pathways, VEGF and ICAM-1.

## Relationship Between Direct and Indirect Effects

Cancer metastasis is closely related to the microenvironment and involves the interaction of surrounding non-cancerous stromal cells, immune system cells (127), extracellular matrix, chemokines, cytokines, and other factors (18). This fragile microenvironment is easily disrupted by surgical procedures; anxiety of surgery creates a window for tumor cells metastasis, leading to immunosuppression, angiogenesis, inflammatory response, and stimulated pain (12). The previous studies also indicated that the development of cancer could be promoted by the inflammatory immune response postoperation for pain and surgical attack (12). Therefore, to investigate the relationship between lidocaine, cancer metastasis, and surgery, ideas for future studies on cancer and anesthetic drugs, as well as for the selection of clinical anesthesia methods and the treatment of cancer are needed.

The cancer microenvironment is extremely complex, with various mechanisms involved. Moreover, as the direct and indirect effects of lidocaine are interactive, they cannot be separated directly. Thus far, clinical studies on the ability of lidocaine to suppress proliferation and induce apoptosis are few, and most of these are relevant to the effects of enhancing pain relief (**Table 1**) (128). So more studies are still required to investigate the specific mechanisms. Data from recent animal and cell studies, to some extent, have explained the proposed mechanisms of lidocaine (**Table 2**), which may be closely correlated to the effects on cancer cell signaling, decreasing immune and inflammatory disruption, and gene modification and instability. Nonetheless, the concentration of lidocaine used in laboratory studies is generally higher than that used in clinical therapy, and lidocaine may be cytotoxic at high concentration (22, 129). Consequently, it may be inappropriate to directly apply the dosage of experimental study to clinical treatment, and it is hard to obtain similar laboratory results in clinical trials. Hence, clinical studies are still needed to determine the appropriate concentration of lidocaine.

## Conclusions

In conclusion, some relevant investigations have demonstrated connections among perioperative events such as lidocaine treatment, surgical stress, and cancer progression. This review summarized and investigated the underlying mechanisms of lidocaine effects on cancer metastasis and recurrence after surgery, and explained the beneficial properties of lidocaine in cancer prognosis which provided some ideas for the clinical treatment and research of cancer. The specific mechanisms of these effects are dependent on further studies.

## Author Contributions

CZ and CX reviewed the literature and drafted the article. YL carefully revised the final manuscript and provided suggestions to improve it. All authors contributed to the article and approved the submitted version.

## Funding

This work is supported by the National Natural Science Foundation of China (No. 81770295) and the Natural Science Foundation of Anhui Province for Outstanding Youth (2008085J34).

## Conflict of Interest

The authors declare that the research was conducted in the absence of any commercial or financial relationships that could be construed as a potential conflict of interest.
